# Clinical features and prognosis for intraventricular liponeurocytoma

**DOI:** 10.18632/oncotarget.16024

**Published:** 2017-03-08

**Authors:** Nini Xu, Jinxiu Cai, Jiang Du, Rong Yang, Huachen Zhu, Peiyi Gao, Jian Zhou, Xiaofeng Li

**Affiliations:** ^1^ Department of Radiology, Beijing Tiantan Hospital, Capital Medical University, Beijing, China; ^2^ Department of Neuropathology, Beijing Neurosurgical Institute, Capital Medical University, Beijing, China; ^3^ PET/CT/MRI Center, The Fourth Affiliated Hospital of Harbin Medical University, Harbin, China; ^4^ Department of Radiology, University of Louisville, Louisville, KY, USA

**Keywords:** cerebellar liponeurocytoma, intraventricular, computed tomography, magnetic resonance imaging, immunohistochemistry

## Abstract

Cerebellar liponeurocytoma is a rare central nervous system tumor, we investigate its biological behaviors and clinical prognosis to improve the understanding of this tumor. We retrospectively analyzed the clinical, radiological and histopathological findings as well as follow-up data of two patients with intraventricular liponeurocytomas in Beijing Tiantan Hospital between July 2000 and July 2016. The main clinical manifestations of the two patients were headache. The supratentorial intraventricular liponeurocytoma appeared as isodense to slight hyperdense on CT scan and heterogeneous intensity on T1-weighted imaging (T1WI) and T2-weighted imaging (T2WI). The plaque-like hypodense on CT images and hyperintensity on T1WI resembling fat could be seen inside the tumor. The liponeurocytoma located in the fourth ventricle showed isointensity on T1 and T2WI as well as slight enhancement on contrast. Two patients accepted gross total resection of tumors. Two intraventricular tumors demonstrated similarly histopathological features, such as isomorphic small tumor cells with clear cytoplasm, sheets of monomorphic round cells and focal lipomatous differentiation. In addition, expression of synaptophysin, neuron specific enolase, microtubule-associated protein 2 and S-100 were found. No radiological or clinical evidence of recurrence of the tumors was observed in their follow-up surveys. In conclusion, intraventricular liponeurocytoma has a favorable clinical course, radiological features may be useful in the diagnosis of this rare tumor before surgery.

## INTRODUCTION

Cerebellar liponeurocytoma is a very rare neoplasm of adults with distinctive morphologic feature of central nervous system (CNS). Some previous cases were reported as unusual tumor in adults under many different names such as lipomatous medulloblastoma, lipidized medulloblastoma and neurolipocytoma. The most accurate description was attributed by Bechtel et al in 1978, they first recognized the salient features of cerebellar liponeurocytoma such as advanced neuronal and focal lipomatous differentiation [1].

The tumor was first introduced to the World Health Organization (WHO) classification in 2000 as a distinct entity under the heading of glioneuronal tumor among the CNS tumors [2]. In the 2007 WHO classification, the tumor was defined as WHO grade II for its low proliferation, but high likelihood of recurrence, and the definition is still in use up to now [3, 4].

This neoplasm develops predominantly in cerebellar hemisphere and vermis, occasionally in supratentorial lateral ventricle and the fourth ventricle. So far, approximately 47 cases of cerebellar, 11 cases of supratentorial intraventricular and 2 cases of fourth ventricular liponeurocytomas are on record in the English literature [5-11]. Comparing to the liponeurocytoma occurred in cerebellum, little is known about intraventricular liponeurocytoma due to the lower number of reported cases. Because of its rarity and paucity of long-term follow-up data, the biological behaviors and clinical features of this tumor are still poorly understood, and there is a lack of agreement on management and prognosis of patients. In recent years, some reports indicated that patients with cerebellar liponeurocytomas usually show a favorable clinical course after neurosurgical removal, it means that the accurate diagnosis of this tumor is very important to avoid unnecessary adjuvant therapy.

Computed tomography (CT) and magnetic resonance imaging (MRI) can clearly show the features of the tumor such as location, size, component and aggression, and these features are very helpful to determine preoperative diagnosis and postoperative recurrence of patients with the tumor.

In this study, we present the clinical, radiological and histopathological features as well as follow-up data of two cases of intraventricular liponeurocytomas, and also review the related literature. We discuss the characteristics of cerebellar liponeurocytoma that may prove useful in identifying this rare tumor.

## RESULTS

**Figure 1:**
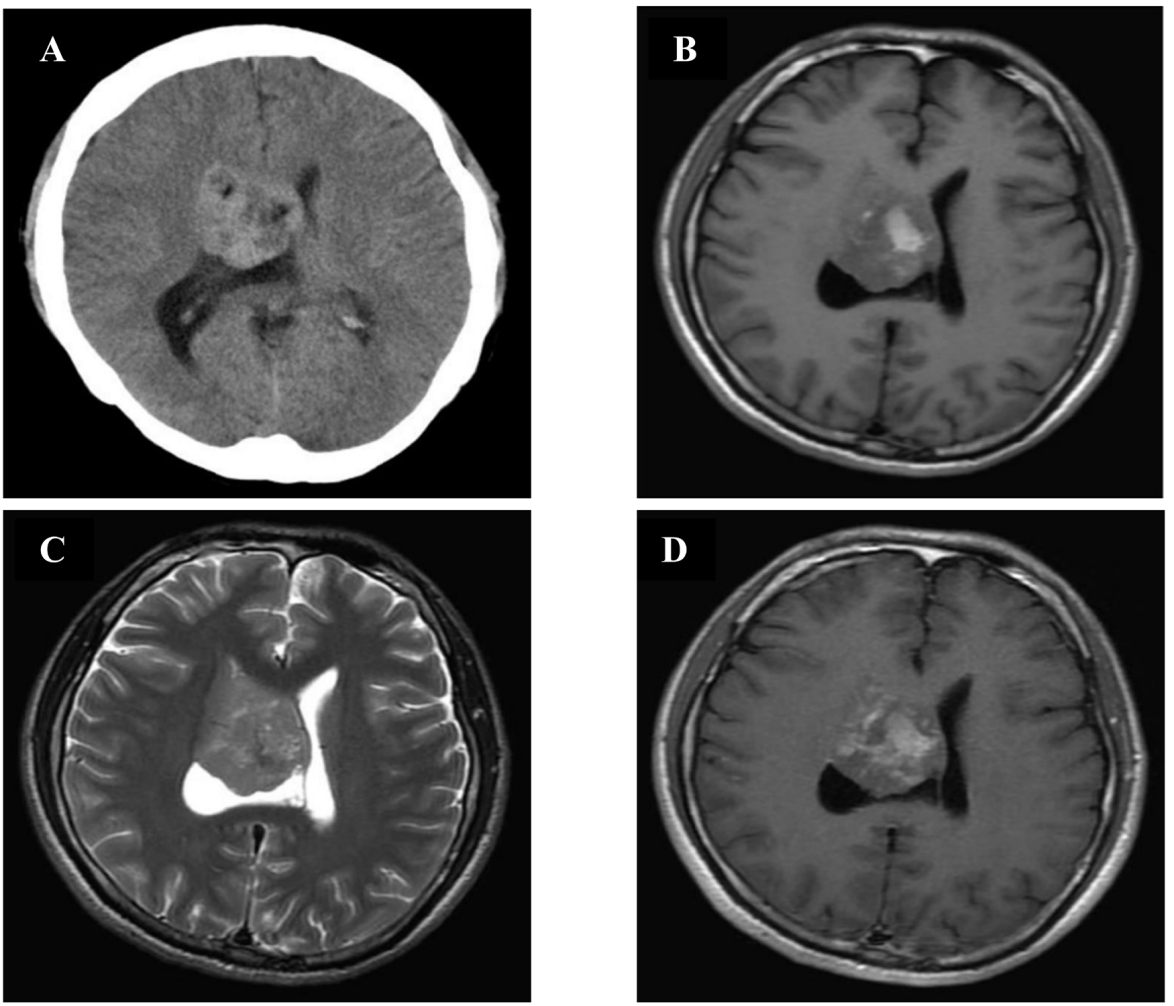
CT and MRI of patient 1 **A.** CT showed a solid lesion in the right lateral ventricle, with fat component (hypodense). (**B.**, **C.**, **D.)** Brain MRI (axial T1WI and T2WI, gadolinium-enhanced T1WI) showed an irregular mass measuring 4.5cm in diameter with a definite boundary in the right lateral ventricle. The mass had heterogeneous components: neuronal (hypo- to isointensity on T1WI and isointensity on T2WI) and fat (strongly T1WI hyperintensity and moderately T2WI hyperintensity). Contrast enhancement of the mass was slight and inhomogeneous.

A 29-year-old man complained of episodic headache for 3 months, progressive aggravation and occasional vomiting. There was no seizure or disorder of consciousness, the patient had normal sensory motor examination. CT scan revealed an isodense to slight hyperdense round mass in the supratentorial right lateral ventricle. The diameter of the mass was approximately 4.5 cm. The lesion was heterogeneous, and an irregular hypodense similar to fat was observed within the lesion (Figure 1A). MRI showed heterogeneous intensity on T1-weighted imaging (T1WI) and T2-weighted imaging (T2WI). The majority of the mass displayed isointensity on T1 and T2 WI as well as high intensity on diffusion-weighted imaging (DWI), and speckled and plaque-like hyperintensity resembling fat could also be seen inside the tumor on T1WI. Mild enhancement was observed after gadolinium administration (Figure 1B-1D). The septum pellucidum was displaced to the left.

The lesion was operated through a right fronto-parietal craniotomy. Under operative microscopy, the mass was mostly located in the front of right lateral ventricle, the base of the mass was situated in the anterior of septum pellucidum, tightly adhering to the thalamus and thalamostriate vein. The mass was yellow-gray, soft suckable, moderately vascular and microcystic. Gross total resection of the tumor was achieved.

**Figure 2:**
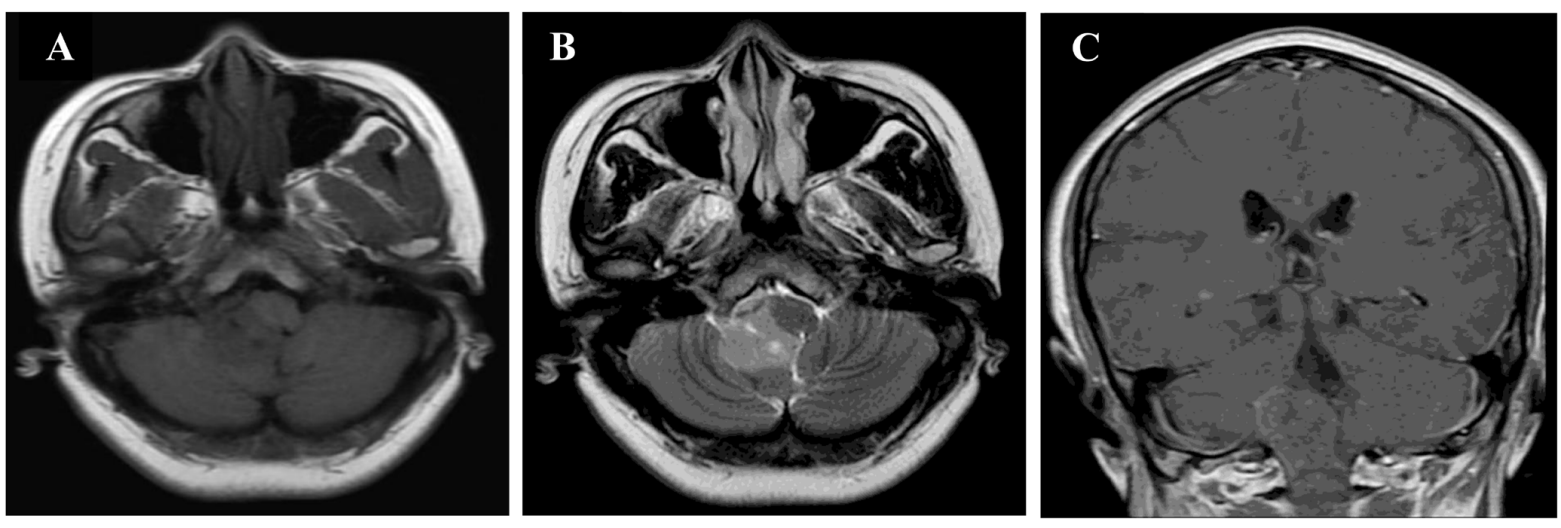
MRI findings in patient 2 **A.**, **B.**, **C.** Brain MRI (axial T1WI and T2WI, coronal T1WI with contrast enhancement). These images showed an irregular lesion filling up the fourth ventricle, and extending down along the cerebellar medullary fissure. The lesion was isointensity on T1 and T2WI with microcysts. Contrast enhancement was slightly observed in the lesion.

The second patient was a 48-year-old woman presented with headache for 1 year, progressive aggravation and numbness of right upper limb. Physical examination showed the decline of superficial sensation of right limbs. MRI revealed an irregular mass with isointensity on T1 and T2WI, several microcysts and slight enhancement on contrast. The mass was located in the fourth ventricle, and involved the right cerebellar hemisphere and lateral foramen of the fourth ventricle (Figure 2A-2C). The mass effect of the lesion was evident, but no surrounding edema was observed.

The neurosurgical excision of the tumor was performed by foramen magnum region craniotomy. The mass was located at the right side of the top of the fourth ventricle, grown down along the cerebellar medullary fissure to the middle of the second cervical vertebral plate, and compressed the medulla oblongata and upper cervical cord. The mass was well-defined, generally yellowish and moderately vascular. Gross total resection of this tumor was achieved.

**Figure 3:**
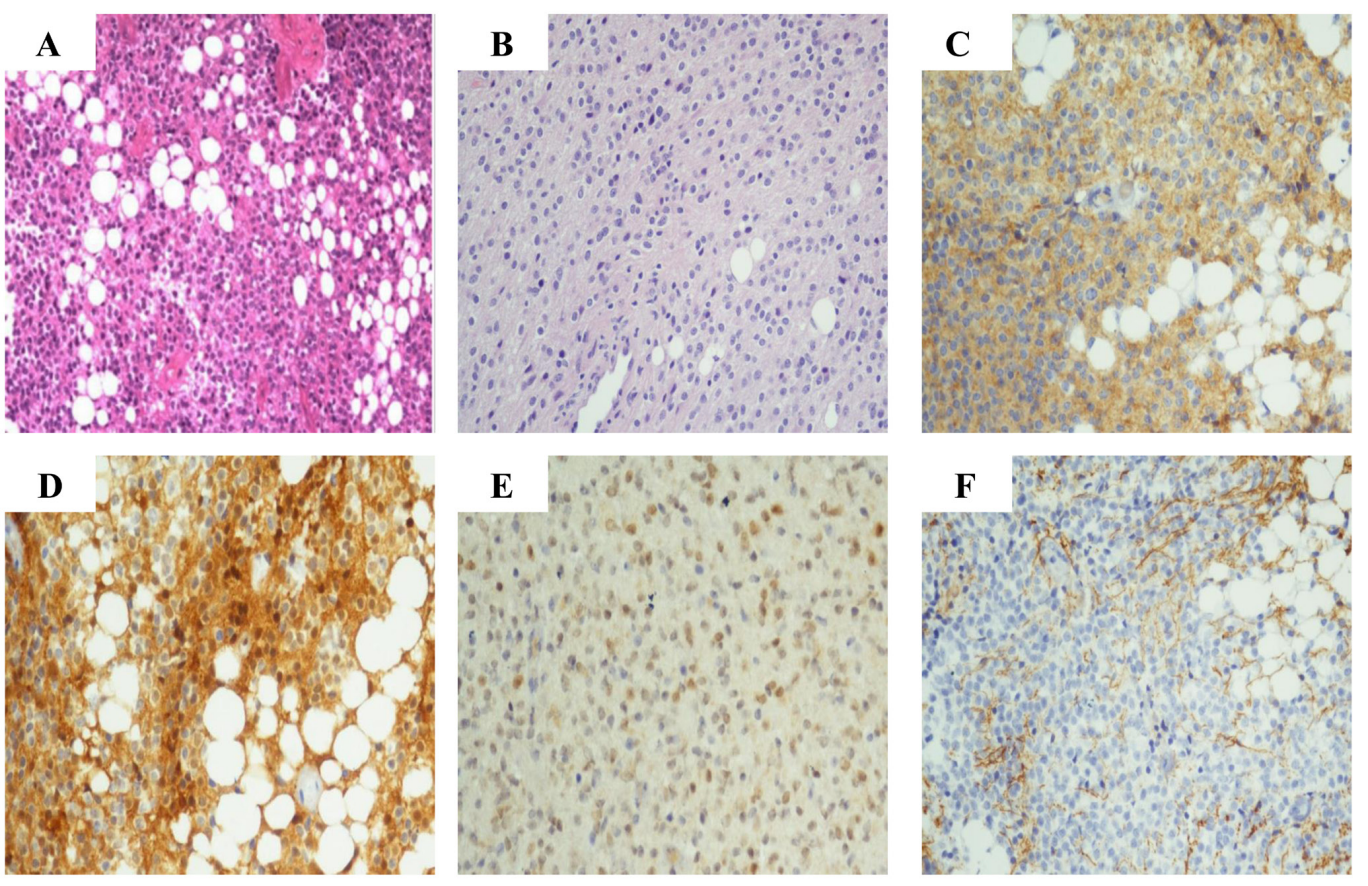
Histopathological and immunohistochemical findings **A.** H&E stained section of case 1 showed sheets of isomorphic tumor cells with round nuclei and clear cytoplasm, and large lipid vacuoles among neoplastic cells. **B.** H&E stained section of case 2 showed sheets of relatively monomorphic tumor cells, neuropil among small tumor cells and some cells containing large intracytoplasmic vacuoles. **C.** Tumor cells were diffuse positivity for synaptophysin in case 1. **D.** Strong labeling was seen with S-100 in tumor cells, lipidized cells and glial cells in case 1. **E.** Tumor cells were moderately positive for NeuN in case 2. F. GFAP was largely negative on tumor cells in case 1. (original magnification × 200)

Histopathological examination showed yellowish coloration, granular calcification and lipomatous tissue in patient 1, and grayish appearance with lipoid tissue in patient 2, on the sections of gross specimen. For two cases, histopathology showed a background of isomorphic small neoplastic cells resembling neurocyte with focal lipomatous differentiation, round to oval hyperchromatic nuclei and clear cytoplasm. Besides the background, the tumors showed respectively the neoplastic cells arrayed intensively with a lot of aggregative cells between the nests of tumor cells in patient 1 (Figure 3A), and the neuropil among tumor cells with occasional adipocyte differentiation in patient 2 (Figure 3B). In the two intraventricular tumors, early and mature neuronal markers such as Synaptophysin (Figure 3C), Neuron specific enolase (NSE), Microtubule-associated protein 2 (MAP-2) and S-100 (Figure 3D) were expressed in neoplastic cells. In patient 2, Neuronal nuclear antigen (NeuN) was immunopositive in tumor cell nuclei (Figure 3E). However, Glial fibrillary acid protein (GFAP) and Neurofilament (NF) were all negative in two patients (Figure 3F). MIB-1 labeling indices were all less than 5 % in two cases (3-4 % for patient 1 and 1-2 % for patient 2). On the basis of these histopathological findings, intraventricular liponeurocytomas were diagnosed in the end.

Both patients were admitted to our institute and received whole tumor resection, there were no adjuvant therapy. In subsequent years, two patients complained of no apparent symptoms. No radiological or clinical evidence of recurrence or residual of the tumor was observed during a nearly 2-year for patient 1 and 5-year for patient 2 follow-up.

## DISCUSSION

Cerebellar liponeurocytoma is a rare central nervous system neoplasm that was initially described as a mixed mesenchymal and neuroectodermal tumor of the cerebellum [1]. The tumor is mainly involved in cerebellar hemisphere and vermis of adults, the peak incidence is from 30 to 60 years old, and the mean age is approximately 53 years. Men and women are affected equally [12]. In the present study, two patients were all adults, but the age of every patient was less than the mean age reported in the medical literature. The main clinical manifestations such as headache and vomiting were usually related to increased intracranial pressure due to mass effect and hydrocephalus.

Cerebellar liponeurocytoma usually appears to be solid mass relative to brain parenchyma on CT and MRI images, the presence of fat inside the tumor revealed on CT and T1WI are the characteristic findings, and the features are very helpful in distinguishing this rare neoplasm from other tumors such as medulloblastoma, ependymoma and central neurocytoma [13]. The hyperintensity on T1WI reflecting fat within the tumor can be suppressed to hypointensity in fat-suppression sequence. This signal intensity change may be useful in determining a preoperative diagnosis of liponeurocytoma. However, we need to clearly recognize that not all the liponeurocytomas can reveal this hyperintensity on T1WI because of different content of fat tissue within the tumor, for example, in patient 2, which the characteristic hyperintensity was not found on T1 and T2WI because of lower fat content inside the tumor. In addition, several microcysts were also clearly displayed on T1 and T2WI. In both tumors, the isodense on CT and isointensity on MRI presented higher cells density. On DWI images, the non-fat component of the tumors revealed mildly restricted diffusion, which reflected the histopathological features of the tumors such as small tumor cells, less cytoplasm and closely arranged tissue cells. On contrast images, the neoplasms showed slightly inhomogeneous enhancement implying mild vascularity in intraventricular liponeurocytomas.

With regard to histopathological features, liponeurocytomas in our cases had biphasic appearance including neurocytic neoplastic cells and focal lipomatous differentiation. The neurocytic tumor cells were arranged in sheet and possessed round to oval nuclei enclosed within clear cytoplasm. The focal lipomatous differentiation presented in clusters or scattered among small tumor cells. These histopathological features were also observed in other cases of intraventricular liponeurocytomas [5, 14]. The tumor cells and lipidized cells of liponeurocytoma may express the neuronal markers such as synaptophysin, NSE and MAP-2. Expression of glial markers like GFAP and S-100 are limited to scattered reactive astrocytes [9, 15, 16]. The immunohistochemical staining in this study showed that the tumor cells experssed synaptophysin, NSE, MAP-2 and S-100 in two cases. MIB-1 labeling index is typically in 1 % to 3 %, but may increase to 5.8 % in the recurrent tumor [5, 15, 17].

Similar to liponeurocytoma occurred in cerebral ventricle, there are few reports on central neurocytoma with lipidized cells [15, 17, 18]. Since central neurocytoma originates from pluripotent progenitor cells in the germinal layers, the presence of fat cells may be acceptable easily [19]. Cerebellar liponeurocytoma located in the fourth ventricle shares some similarities with medulloblastoma in terms of location and cells of origin. However, the genetic analysis has concluded that cerebellar liponeurocytoma as a distinct entity is different from medulloblastoma because of a higher frequency of TP 53 missense mutation than medulloblastoma [16, 20]. Moreover, isochromosome 17q, a genetic hallmark of cerebellar medulloblastoma was not observed in any of the cases investigated about cerebellar liponeurocytoma study [6, 16, 20].

Cerebellar liponeurocytoma generally has a favorable clinical course for its benign biological behaviors. However, the increased risk of recurrence should be taken into account when mentioned the atypical liponeurocytoma. It has been reported that the recurrence rate of cerebellar liponeurocytoma was about 31% [21]. From the view of clinical management, the best treatment of cerebellar liponeurocytoma is completely surgical removal, and repeat surgery may be preferable to radiotherapy for recurrent liponeurocytoma. But it is unclear whether radiotherapy should be given for recurrent tumor occurred after many years of initial neurosurgery [12, 22]. The related reports suggested that the 5-year-survival rate of cerebellar liponeurocytoma was 48 %, but this should be interpreted with caution because of the paucity of long-term follow-up data [3, 5]. In the present study, the two patients underwent gross total resection of the tumors, and were not treated with adjuvant therapy. No neurological evidence of recurrence or residual of the tumors was observed at outpatient serial follow-ups. As for postoperative follow-up, the interval from initially surgical removal to the last recheck was different from ten months to two years and six months in Chinese patients with liponeurocytomas, and most of the cases were lost to follow-up in the interval of one year following initial neurosurgery (Table 1).

**Table 1:** Clinical summary of ten reported Chinese cases of cerebellar liponeurocytomas

Authers	Age/	Clinical	Site	Imaging findings	Treatment	Prognosis
	Sex	Features				
Sun et al.	43y/F	Dizziness	Cerebeller	MRI: isointense on T_1_WI and T_2_WI,	Gross total	Not mentioned
		6 days	vermis	brilliantly enhancing on contrast	recection	
Guan et al.	50y/M	Headache	Cerebeller	MRI: isointense lesion on T_1_ and T_2_WI,	Gross	Not mentioned
		2 months	vermis	hyperintense can be seen on T_1_WI, mildly and inhomogenously enhancing on contrast	recection	
Liu et al.	44y/F	Headache	Cerebeller	MRI: iso-to hypointense on T_1_WI and iso-to	Gross	Not mentioned
		dizziness	vermis	hyperintense on T_2_WI, enhanced mildly and	recection	
		3 months		specky on contrast	postoperative radiotherapy	
Shu et al	27y/M	Headache	Cerebeller	MRI: slightly hypointense on T_1_WI and	Gross total	No recurrence at
		2 weeks	vermis	hyperintense on T_2_WI	recection	1-year, and the
					postoperative radiotherapy	patient was lost for follow-up
Wang et al	47y/F	Headache	Cerebeller	CT: isodense leision with central patches of	Gross total	Not mentioned
		dizziness	hemisphere	hypotense. MRI: hypointense on T_1_ WI, and	recection	
		6 months		hyperintense on T_2_WI, short T_1_ hyperintense on T_1_WI, mildly enhancing on contrast		
Qin et al.	63y/M	Headache	Cerebeller	CT: isodense leision and hypotense in center.	Gross total	Not mentioned
		dizziness	hemisphere	MRI: hypointense on T_1_ and hyperintense	recection	
		2 months		on T_2_WI, short T_1_ hyperintense on T_1_WI		
Yang et al.	54y/F	Headache	Cerebeller	MRI: slightly hypointense on T_1_WI, and	Gross total	No recurrence
		dizziness	hemisphere	isointense on T_2_WI, brightly short T_1_ area on	recection	at 18-month,
		1 year		T_1_WI, inhomogenously enhancing on contrast		and the patient was lost for follow-up
Wang et al	30y/M	Headache	Right	CT: slightly hypodense round leision with	Gross total	Not mentioned
		dizziness	lateral	small cystic areas	excision	
		1 months	ventricle			
Shi et al.	36y/M	Headache	Left lateral	CT: hypo-to isodense mass leision. MRI:	Gross	No recurrence at
		dizziness	ventricle	isointense on T_1_WI and T_2_WI, short T_1_	recection	2.5-year, and the
		12 months		hyperintense displayed on T_1_WI	postoperative radiotherapy	patient was lost for follow-up
Ding et al.	52y/F	Headache	Third	CT: round hypodense leision with dark area	Gross total	No recurrence
		10 days	ventricle	suggestive of fatty tissue, and also speckled calcification	recection	at 10-month, and the patient was lost for follow-up

In summary, intraventricular liponeurocytoma has distinctive morphologic and immunophenotypic features. The characteristic findings corresponding to fat content inside tumor on CT and MRI may be useful in differentiating this rare tumor from other neoplasms before surgery. Larger studies based on long-term follow-up are necessary to elucidate the clinical features and prognosis of cerebellar liponeurocytoma.

## MATERIALS AND METHODS

### Patients

This study was pre-approved by Beijing Tiantan Hospital Institutional Review Boards. We reviewed our records at the Department of Radiology and Neuropathology, Beijing Tiantan Hospital, a tertiary medical institution specialized for neurological diseases, over a period of 16 years from July 2000 to July 2016. Two cases of adult intraventricular liponeurocytomas defined by routine histopathological and immunohistochemical examinations after neurosurgical operation were found. One occurred in supratentorial lateral ventricle and the other located in the fourth ventricle. We reviewed the clinical, radiological and histopathological features, and contacted with the patients and their family members to get their serial follow-up data.

### CT and MRI protocol

CT scan was performed with a Somatom Sensation 16 CT scanner (Siemens Medical System, Germany). Scanning was conducted with 120 kVp, 310 mAs, field of view = 228, matrix = 512 × 512, and 5 mm slice thickness. MRI data were acquired on a 1.5 T MRI system (Signa HDe, GE healthcare, USA). MR images were collected with the following parameters: axial T1WI FLAIR (repetition time = 2379 ms, echo time = 9.8 ms) and T2WI (repetition time = 5260 ms, echo time = 104 ms), field of view = 240 mm × 240 mm, matrix = 512 × 512, slice thickness = 5 mm, and four averages. A bolus of 4 mL/s of gadolinium-diethylene-triamine pentaacetic acid (0.1 mmol/kg, Bellona, Beijing, China) was injected through an *elbow* vein cannula, and contrast-enhanced T1WI was obtained.

### Histology and immunohistochemistry assay

The excised tumor tissues were routinely processed. The sections were stained with hematoxylin and eosin (H&E) for routine histopathological evaluation. Immunohistochemical examinations were also conducted on representative sections using indirect immunoperoxidase technique with antibodies to NSE (1:100; Dako USA), GFAP (1:50; BioGenex USA), Synaptophysin (1:50; Dako USA), NeuN (1:50; BioGenex USA), S-100 (1:100; BioGenex USA), NF (1:1000; BioGenex USA), MAP-2 (1:1000; Sternberger Monoclonals USA) and MIB-1 (1:50; Dako USA).
